# Spectrum of Injury Presenting to Emergency Department in A Tertiary Care Hospital in Nepal: A Descriptive Cross-sectional Study

**DOI:** 10.31729/jnma.5248

**Published:** 2020-09-30

**Authors:** Sameer Thapa, Anup Raj Upreti, Bishwa Raj Dawadi

**Affiliations:** 1 Department of Emergency Medicine and General Practice, Nepal Medical College and Teaching Hospital, Attarkhel, Kathmandu, Nepal; 2 Clinical Pharmacist, Nepal Medical College and Teaching Hospital, Attarkhel, Kathmandu, Nepal; 3 Department of Emergency Medicine and General Practice, Grande International Hospital, Dhapasi, Kathmandu, Nepal

**Keywords:** *fall*, *injury*, *road traffic accident*

## Abstract

**Introduction:**

Injury is one of the major global public health problems causing significant number of death and disability. The study aims to study the epidemiological and clinical profile of patients presented in emergency department with injury.

**Methods:**

This was a descriptive cross-sectional study conducted in a tertiary care hospital from September 2019 to February 2020 after obtaining ethical approval from Institutional review board (reference number 007-076/077). A convenient sampling method was applied. Epidemiological factors, chronological factors, causes of injury, anatomical distribution, pattern of injury were studied. Statistical analysis was done using statistical package for the social sciences version 20. Point estimate at 95% Confidence Interval was calculated along with frequency and proportion for binary data.

**Results:**

Out of 197 patients, 72 (36.5%) patients had fall followed by road traffic accident 57 (28.9%). Of total, 80 (40.6%) had injury at home and 80 (40.6%) had cut injury. Head and neck accounted for 66 (33.5%) of total injury followed by upper Extremities 50 (25.4%) and lower extremities 47 (23.9%). Eighty-seven (44.2%) of the patients visited emergency within 30 minutes of sustained injury.

**Conclusions:**

The top three leading causes of injuries visiting emergency department were: fall, Road Traffic Accident and physical assaults respectively. The most common mode was fall being cut as most common pattern. Head and neck was the most commonest site of injury. The common place of injury was home.

## INTRODUCTION

Injuries is a most important global public health challenges causing significant mortality and permanent disability.^1^ The burden of injuries is gradually rising and more devastating in developing countries^2,3^ due to rapid urbanization,industrialization,changing in life style, poorly built infrastructure, non maintained road system and limited prehospital and emergency care.^4^

Rising trends of Injuries due to violence and accident accounts for about 8% of death in Nepal.^4^ While injury can be preventable,^5^ evaluation of different dimension of injured patients presenting to emergency department support to develop and promote preventive measures and formulate clinical guidelines in the management of injured patient.

In this study, we have attempted to study about the demographic variables along with distribution, mode and patterns of injury of patients presented in emergency department.

## METHODS

This descriptive cross-sectional study was conducted in Nepal Medical College Private Limited and Teaching Hospital (NMCTH) for a period of six months (September 2019 to February 2020). Ethical clearance was obtained from the Nepal Medical College Institutional Review Committee (reference number 007-076/077). Patients presenting to the emergency department with injury were enrolled in the study (n= 197).

The sample size was calculated as,

$$\begin{array}{ll} \text{n} & \text{= }{\text{Z}}^{\text{2}}\times \text{p}\times \text{q}/{\text{e}}^{\text{2}}\\ & \text{= }{\left(1.96\right)}^{\text{2}}\times (0.15)\times (1-0.15)/{\left(0.05\right)}^{\text{2}}\\ & \text{= }195.84\\ & =196 \end{array}$$

where,

n = sample size
p = prevalence of injury, 15%
e = margin of error, 5%
Z = 1.96 at 95% Confidence Interval

All cases of injury irrespective of age, sex, mode, type, place anatomical distributioin, pattern and the status of patients, after injury were included in the study. People from whom history could not be obtained and those not willing to participate were exclusion criteria in this study.

Written consent was obtained from participants. Epidemiological factors like age, gender, educational status, occupation, marital status, religion, and ethnicity were studied. Chronological factors like arrival time, time elapses in presentation to hospital were also included in the study. Etiology, anatomical distribution, pattern of injury and outcome was also studied. A convenient sampling method was applied.

The collected data were analyzed in the Statistical Package of the Social Sciences version 20 by using descriptive statistics. Point estimate at 95% Confidence Interval was calculated along with frequency and proportion for binary data.

## RESULTS

The mean age of the patients was 26.62±18.69 years with minimum age of 1 years and maximum age of 82 years. Majority of the patients were male 134 (68%) and unmarried 107 (54.3%). Majorities were from Hindu religion 131 (66.5%), followed by Buddhist 62 (31.5%), Christian 2 (1%) and Islam 2 (1%). Out of 197 patients, 65 were students (33%), 37 were service holders (18.7%), 23 were housewife (11.7%), 22 were laborer (11.2%), 14 were business holders (7.1%), 8 were unemployed (4%), 7 were farmer (3.6%), 3 were driver (1.5%) and remaining 18 were unknown (9.18%). 56 were illiterate (28.4%) and majority have completed primary level of education (34.5%, n= 68) followed by secondary level of education 36 (18.3%), Plus 2 17 (8.6%), bachelors 11 (5.6%), SEE 9 (4.6%). Regarding alcohol use, only 45 were under influence of alcohol (22.8%). One hundred fifty-nine had external injury (80.7%).

The most common cause of injury was fall 72 (36.5%) followed by road traffic accidents 57 (28.9%) (Table 1).

**Table 1:** Etiology of Injury (n = 197).

	n (%)
Fall	72 (36.5)
Road Traffic Accident	57 (28.9)
Physical Assault	26 (13.2)
Self-Inflicted	9 (4.6)
Machinery	7 (3.6)
Others	26(13.2)
Total	197 (100.0)

Home was the commonest place of injury 80 (40.6%) followed by road 75 (38.1 %) (Table 2).

**Table 2:** Place of Injury (n = 197)

	n (%)
Home	80 (40.6)
Road	75 (38.1)
Work Place	24 (12.2)
School	12 (6.1)
Inside Vehicle	1 (0.5)
Others	5 (2.5)
Total	197 (100.0)

Head and neck 66 (33.5%) was the most common anatomical site of injury followed by Upper Extremities 50 (25.4%) (Table 3).

**Table 3:** Anatomical Distribution of Injury (n = 197).

Anatomical Distribution	n (%)
Head and Neck	66 (33.5)
Upper Extremities	50 (25.4)
Lower Extremities	47 (23.9)
Face	23 (11.7)
Pelvic and Groin	3 (1.5)
Spine	3 (1.5)
Chest & Thorax	3 (1.5)
Abdomen	2 (1.0)
Total	197 (100.0)

Similarly, Cut was the most common pattern of injury 80 (40.6%) followed by laceration 38 (19.2%) (Table 4).

**Table 4:** Pattern of injury on body regions (n=197).

	n (%)
Cut	80 (40.6)
Laceration	38 (19.2)
Fracture	30 (15.2)
Abrasion	24 (12.18)
Bruise	16 (8.12)
Dislocation	9 (4.5)
Total	197 (100.0)

Most patients visited emergency department within 30 minutes of trauma 87 (44.2%) (Table 5).

**Table 5:** Interval between Trauma and Arrival (n=197).

	n (%)
<30 Min	87 (44.2)
>2 Hrs	32 (16.2)
1-2 Hrs	25 (12.7)
30-60 Min	53 (26.9)
Total	197 (100.0)

One hundred twenty were discharge from emergency department (60.9%) and 30 left against medical advice (15.25%) (Table 6).

**Table 6:** Outcome of Patients (n = 197).

Outcome	n (%)
Discharge	120 (60.9)
Leave Against Medical Advice	30 (15.2)
Admitted	21 (10.7)
Refer	13 (6.5)
Death	7 (3.6)
Absconded	6 (3.0)
Total	197 (100.0)

## DISCUSSION

In our study the mean age was found to be 26.62 years while in the study done in Africa, the mean age of the patients was 30.2 years.^6^ There was predominance of male gender in our study which was similar to studies done in Nepal.^7,8^ Majority were Hindu. Students were more prone to injury in our study which was similar to the study done in Africa.^6^ Most of them were illiterate in our study. Nonalcoholic were almost thrice as common as alcoholic (Alcoholic: Nonalcoholic: 1: 3.3). In a systematic review article to investigate the epidemiology of road traffic injury (RTI) in Nepal, out of 11 only 3-article mention alcohol consumption as a possible causes of accidents.^9^ Majorities of the patients had some forms of external injury which was similar to the study done in Nepal.^7^

The most common cause of trauma was fall followed by road traffic accident which was similar to the studies done in Nepal.^7,10^ In a systematic review study of injuries and violence related articles in Nepal, falls was the most common cause of neuro trauma whereas road traffic injuries were the most common form of injuries.^11^ In a fall injuries survey done in Nepal, falls represented 37.2% of total injuries.^12^ In our study falls accounted for 36.5%. Home was the commonest place of injury which was similar to the study done by Verma S, et al^13^ in pediatric populations. Injuries to head and neck (33.5%) were the commonest ones followed by injury to upper extremities (25.4%). In total, injuries to extremities accounted 49.2% (n= 97). Injury to extremities were the commonest ones followed by injuries to head and neck (13.9%) in the study done by Shrestha R, et al.^7^

In our study, cut was the most common pattern of injury followed by laceration and fracture. Cuts and open wounds accounted for 40.2% of all injuries in the study done by Joshi SK.^8^ In another study done by Joshi SK, et al. open wounds including incised and lacerated ones constitute the most common form of injury.^11^

Most of them came to emergency department within 30 min (44.2%). More than 60% patient visited emergency department within an hour.

Majority, almost two third, of the patients presenting to the hospital were discharged after appropriate management in the emergency department which was similar to the study done by Shrestha R, et al.^7^ However, 15.2% (n = 30) left the emergency department against medical advices.

## CONCLUSIONS

The three top most leading causes of injuries visiting emergency department were: fall, road traffic accident and physical assaults respectively. The most common mode was fall being cut as most common pattern. Head and neck was the most commonest site of injury. The common place of injury was home. Most of them visited emergency department within 30 minutes of trauma. Hospital-based single-center study and limited sample size should be considered as the limitation of this study. Therefore, a multi-center study with a large sample size should be considered as these kind of data provide an insight to the magnitude of different types of injuries in tertiary level hospital, and hence will be helpful in guideline and protocol formulation.
